# Effect of depression on mortality and cardiovascular morbidity in type 2 diabetes mellitus after 3 years follow up. The DIADEMA study protocol

**DOI:** 10.1186/1471-244X-12-95

**Published:** 2012-07-30

**Authors:** Carmen de Burgos-Lunar, Paloma Gómez-Campelo, Juan Cárdenas-Valladolid, Carmen Y Fuentes-Rodríguez, María I Granados-Menéndez, Francisco López-López, Miguel A Salinero-Fort

**Affiliations:** 1 Unidad de Epidemiología Clínica e Investigación, Hospital Carlos III, (C/ Sinesio Delgado, 10), Madrid, (28029), Spain; 2 Fundación de Investigación Biomédica, Hospital Carlos III, (C/ Sinesio Delgado, 10), Madrid, (28029), Spain; 3 Unidad de Apoyo Técnico, Gerencia Adjunta de Planificación y Calidad del Servicio Madrileño de Salud, (C/ O’Donell, 55), Madrid, (28007), Spain; 4 Gerencia, Hospital Carlos III, (C/ Sinesio Delgado, 10), Madrid, (28029), Spain; 5 Centro de Salud Monóvar, Dirección Asistencial Este, Servicio Madrileño de Salud, Madrid, Spain; 6 Centro de Salud Vicente Muzas, Dirección Asistencial Este, Servicio Madrileño de Salud, Madrid, Spain

**Keywords:** Depression, Diabetes mellitus, Type 2, Primary health care

## Abstract

**Background:**

Type 2 diabetes mellitus and depression are highly prevalent diseases that are associated with an increased risk of cardiovascular disease and mortality. There is evidence about a bidirectional association between depressive symptoms and type 2 diabetes mellitus. However, prognostic implications of the joint effects of these two diseases on cardiovascular morbidity and mortality are not well-known.

**Method/design:**

A three-year, observational, prospective, cohort study, carried out in Primary Health Care Centres in Madrid (Spain). The project aims to analyze the effect of depression on cardiovascular events, all-cause and cardiovascular mortality in patients with type 2 diabetes mellitus, and to estimate a clinical predictive model of depression in these patients.

The number of patients required is 3255, all them with type 2 diabetes mellitus, older than 18 years, who regularly visit their Primary Health Care Centres and agree to participate. They are chosen by simple random sampling from the list of patients with type 2 diabetes mellitus of each general practitioner.

The main outcome measures are all-cause and cardiovascular mortality and cardiovascular morbidity; and exposure variable is the major depressive disorder.

There will be a comparison between depressed and not depressed patients in all-cause mortality, cardiovascular mortality, coronary artery disease and stroke using the Chi-squared test. Logistic regression with random effects will be used to adjust for prognostic factors. Confounding factors that might alter the effect recorded will be taken into account in this analysis. To assess the effect of depression on the mortality, a survival analysis will be used comparing the two groups using the log-rank test. The control of potential confounding variables will be performed by the construction of a Cox regression model.

**Discussion:**

Our study’s main contribution is to evaluate the increase in the risk of cardiovascular morbidity and mortality, in depressed Spanish adults with type 2 diabetes mellitus attended in Primary Health Care Setting. It would also be useful to identify subgroups of patients for which the interventions could be more beneficial.

## Background

Type 2 diabetes mellitus (T2DM) is a highly prevalent chronic disease which affects more than 10% of the adult population of the developed countries [1,2], and between 70% and 80% of people with diabetes die as a result of cardiovascular complications [3-7].

Depression is also highly prevalent, approximately 5.8% of men and 9.5% of women will experience a depressive episode in a year [8]. There is also evidence to suggest that depression is associated with a significantly increased risk of cardiovascular disease [9] and all-cause mortality [10].

The prevalence of depression in patients with T2DM is near twice greater than for non-diabetic subjects [11,12]. There is a strong body of evidence for the association of T2DM with depression [11-13], and both diseases are associated with unemployment and problems with work performance [14].

However, data evaluating prognostic implications of the joint effects of these two diseases on cardiovascular disease and mortality are sparse.

### Depression and diabetes: bidirectional relationship

Although there is ample evidence about a bidirectional association between depressive symptoms and T2DM [15], there is a controversial gap in the direction of the cause-effect [16,17], and, also the casual factors that are intermediate in the relationship remain unclear [18].

Several studies have examined whether depression is a predictor for the onset of T2DM, most of which confirm an increase of risk of T2DM in depressed patients, but differ in the extent of this increase that ranges between 32% and 60% [15,17,19,20].

On the other hand, several studies assessed if the T2DM may also increase the risk for depression. In the absence of comorbidities and complications, the data showed relationship between T2DM and the onset of depression [15-17]; with an incidence estimate among 15% to 24% [15,16,18]; after controlling for potential confounders factors, the T2DM is associated with an increased risk of depression (Odds Ratio (OR) = 1.41) [20].

### Clinical and socioeconomic impact of the depression in patients with DM2

The coexistence of depression and diabetes is associated with poor adherence to treatment, dietary and weight loss recommendations and most frequently present other cardiovascular risk factors such as smoking, obesity, sedentary lifestyle and poor glycemic control, present a reduced health related quality of life (HRQoL), disability and increased health care expenditures, than T2DM patients without depression [10,21-24].

Previous studies have shown that comorbid depression in diabetic patients was associated with a significant increase of risk of macrovascular complications (OR: 2.64) [25], microvascular complications (OR: 11.32) [23] and is associated with an increase in risk of all-cause mortality (OR: 1.33-1.52), after controlling for sociodemographic factors and clinical severity of illness [26-30].

Thus, there is an additional increase in health-service costs of 50% in diabetes with comorbid depression [9].

### Predictors of depression in T2DM patients

Few studies have been conducted to develop and validate predictive models of depression in patients with T2DM. The Pathways Epidemiology Study [31] showed that the risk of major depression among patients with T2DM is increased by depression history, baseline diabetes symptoms, and previous cardiovascular disease. Recently, the results of a small study with 90 outpatients, which examine psychosocial and clinical variables associated with depression in T2DM patients, showed that depression in diabetic patients was predicted by diabetes related distress, life events and neuropathy [32]. In the predictD Study, King and colleagues elaborated a risk algorithm for depression that included different variables such as age, sex, educational level, personal and family history of psychological difficulties, HRQoL, and social support, among others [33]. However, frequently, studies in this research area not included the factors identified in the predictD Study for onset of depression in adult general population.

Until now, the mechanisms linking depression and T2DM are still not sufficiently known.

In sum, there is evidence that depression is associated with T2DM, and this interaction increased mortality, complications, and disability, as well as an earlier occurrence of all these adverse outcomes. However, previous investigations have been limited by the use of self-reported scales or data from National Health Surveys, with a limited value as diagnostic tool. The mechanisms linking these diseases are not well-known and it would be useful to have a predictive clinical model to emphasize on T2DM patients more vulnerable for depression, which would enable intervention in a timely manner to prevent or treat early those patients.

### Main objectives

1.To analyze the effect of depression on cardiovascular events (acute myocardial infarction and stroke), all-cause and cardiovascular mortality in patients with T2DM after 3 years follow-up.

2.To estimate a clinical predictive model of depression in patients with T2DM.

### Secondary objective

To determine the prevalence and the incidence rate, crude and standardized, by age and sex, of depression in patients with T2DM.

## Methods/design

### Design

A three-year, observational, prospective, cohort study, carried out from January 1^st^ 2011 to 31^th^ December 2013.

### Setting

The study will be carried out in 75 Primary Health Care Centers (PHCC) in the region of Madrid, Spain.

### Subjects of the study

Subjects with diagnosis of T2DM in their computerized clinical records (CCR) and are usually monitored by their process in PHCC.

All PHCC in Madrid have CCR available since 2003 and the diagnoses of diabetes recorded in these have been validated [34].

### Inclusion criteria

18 years of age or older at the date of January 1^st^ 2011.

Patients who had visited their PHCC at least twice in the last year.

Agree to participate in the study and written informed consent.

### Exclusion criteria

Diagnosis of gestational diabetes mellitus.

Institutionalized patients.

Subjects who cannot understand Spanish.

Patients with severe chronic diseases or significant physical or psychic disabilities that might invalidate informed consent or interview (according to clinical judgment).

Legal incompetence or legal guardianship.

Participation in clinical trials.

### Sample size

#### Method of calculation

For the first main objective, for an alpha of 0.05, a power of 80%, a prevalence of depression of 17.6% [13] and in order to detect an increase of 25% [25] in the mortality of depressed patients, the overall sample size required 2959 patients. Given these assumptions, and expecting a 10% loss rate, the final sample size required was 3255 patients.

The sample size considerations regarding mortality are based on the results of Schramm in a 5-year follow-up study [35], corrected for a 3-year period.

The sample size required to perform the other objectives of the study is lower, so the sample size estimated enables to address all the above objectives.

### Randomization

Patients with T2DM will be selected by simple random way from the patients with T2DM of each participating general practitioner.

During consultations, patients will be informed about the study and asked whether they would like to take part in it. Those who accept will be asked to complete a signed consent form. Checks will be made to ensure they met all inclusion criteria, but no exclusion criterion.

### Variables

The main outcome measures are all-cause and cardiovascular-specific mortality and cardiovascular morbidity (acute myocardial infarction, and stroke).

The exposure variable is the major depressive disorder.

Sociodemographic variables: age (date of birth), gender, nationality, time of residence in Spain, marital status (single, unmarried partners, married, divorced, widowed), educational level (no studies, primary, high school, university), employment status (working, working with a low of three months or more, unemployed, retired/pensioner, student, housework and other situation), employment (manager with over 10 employees, managers with fewer than 10 employees, administrative, self-employed workers and supervisors, skilled manual, semiskilled manual and unskilled manual) and social class (I-V) [36].

Comorbidity variables: hypertension, heart failure, retinopathynephropathy, neuropathy, amputations, erectile dysfunction, and renal failure.

Other clinical variables: family history (in the first-degree relatives) of diabetes, diabetes duration, and sleeps quantity and quality.

Anthropometric variables: height, weight, hip circumference, waist circumference, systolic blood pressure, and diastolic blood pressure.

Laboratory results: albuminuria, creatinine, lipid profile, A1C and glucose.

Personal health habits: smoking (never, former or current smoker), physical activity level (sedentary, moderate-intensity, vigorous-intensity, competition-level), and drinking (0.1 through 4.9, or 5.0 or more g/d of alcohol).

Treatment: statins, β-blockers, angiotensin-converting enzyme inhibitors, angiotensin receptor blockers, calcium channel blockers, diuretics, antiplatelets, antidiabetics, antidepressant drugs and anxiolytics which were prescribed.

Psychosocial variables: family history (in the first-degree relatives) of psychiatric disorder, personal history of psychiatric disorder, previous and current therapeutic plan, current generalized anxiety disorder, social support, and HRQoL.

The variables and their instrument of measurement are summarized in Table 1.

**Table 1:** Variables and measurement instruments

**Endpoint**	**Measuring instrument**	**Sources**	**Baseline**	**Year 1**	**Year 2**	**Year 3**
**Cardiovascular events**	eCRF completed with the CCR	General practitioners	✓	✓	✓	✓
**Mortality and cause of death**	Death certificate Family reported	General practitioners, National Death Index Interview by psychologist	✓	✓	✓	✓
**Major depressive disorder**	MINI	Interview by psychologist		✓	✓	✓
**Generalized anxiety disorder**	MINI	Interview by psychologist		✓	✓	✓
**Sociodemographic variables**	Self-report questionnaire	Interview by psychologist	✓			
**Personal health habits**	Self-report	Interview by psychologist	✓	✓	✓	✓
**Anthropometric variables**	eCRF completed with the CCR	General practitioners	✓	✓	✓	✓
**Comorbidity and complications**	eCRF completed with the CCR	General practitioners	✓	✓	✓	✓
**Laboratory results**	eCRF completed with the CCR	General practitioners	✓	✓	✓	✓
**Treatment**	eCRF completed with the CCR	General practitioners	✓	✓	✓	✓
**Sleep quantity**	Self-report	Interview by psychologist	✓	✓	✓	✓
**Sleep quality**	AIS	Interview by psychologist	✓	✓	✓	✓
**Personal and family history of psychiatric disorder**	Self-report	Interview by psychologist	✓			
	CCR	General practitioners				
**Social support**	First item of RSE	Interview by psychologist		✓	✓	✓
**HRQoL**	SF-12	Interview by psychologist		✓	✓	✓

*eCRF*: electronic case report forms; *CCR*: computerized clinical records; *MINI*: Mini-International Neuropsychiatric Interview; *AIS*: Athens Insomnia Scale ; *SF-12*: Short-Form Health Survey.

### Data collection method

**Figure 1:**
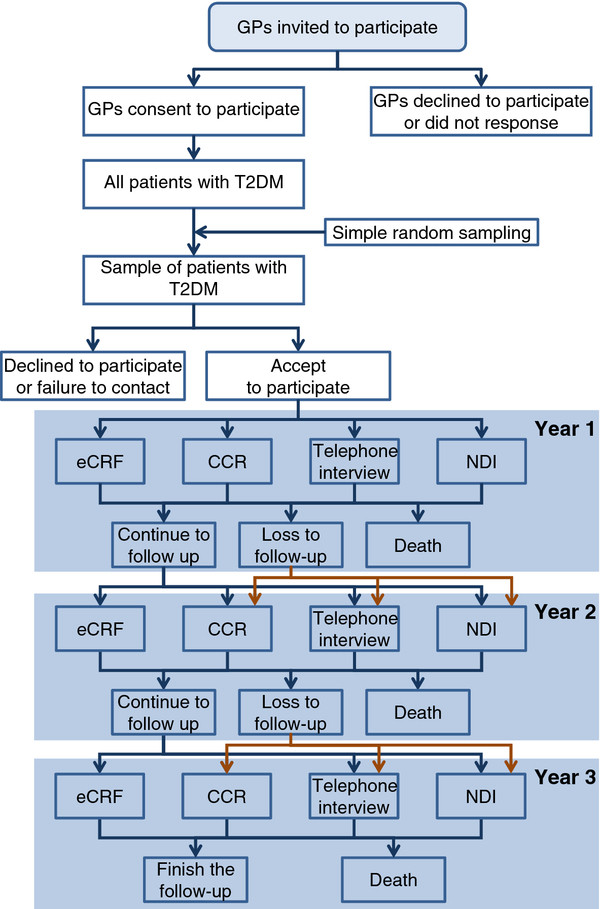
**Flowchart.** GPs: General Practitioners; T2DM: Type 2 diabetes mellitus; eCRF: electronic case report forms; CCR: computerized clinical records; NDI: National Death Index

The Figure 1 shows the flowchart of the study.

### General practitioners

Medical evaluation will be performed under normal clinical practice conditions, and clinical variables, anthropometric measures, treatments and laboratory results will be collected by general practitioners at baseline and annually during follow-up. These data will be recorded in an electronic Case Report Forms (eCRF) hosted in the website http://www.madiabetes.com.

### Clinical psychologist interview

Different questionnaires and a psychological evaluation will be conducted by clinical psychologists at baseline and annually during follow-up through a telephone interview. The following variables will be collected by this way: current major depressive disorder, generalized anxiety disorder, personal and family history of psychiatric disorder, sociodemographic variables, social support, HRQoL, sleep quantity and quality.

The conversation protocol will be designed in advance and the interviewers will receive homogeneous training in the evaluation procedure of the study in order to minimize the variability in the data collection.

The diagnosis of a current major depressive disorder will be conducted through a semi-structured interview to detect those who meet the Diagnostic and Statistical Manual of Mental Disorders-Fourth Edition (DSM-IV) criteria (codes: 296.26-299.20) [37]. To meet criteria for depression patients it is required to have at least 5 symptoms, including at least one of the cardinal symptoms: depressed mood or anhedonia, during two-week period. The interview will be based on the module of major depressive disorder of the Mini-International Neuropsychiatric Interview (MINI) [38], and according to clinical judgment. The use of antidepressant medication in previous three months will also be used as a measure of depression.

The diagnosis of current generalized anxiety disorder will be performed by a semi-structured interview to detect those who meet the DSM-IV criteria (code 300.02). To meet criteria for this disorder, patients required to have at least 3 of the central symptoms of anxiety (feeling wound-up, tense, or restless easily becoming fatigued or worn-out, concentration problems, irritability, significant tension in muscles or difficulty with sleep), at least 6 months. The interview will be based on the module of generalized anxiety disorder of the M.I.N.I. [38] and according to clinical judgment.

The MINI is a short and efficient diagnostic interview to diagnose mental disorders, compatible with International diagnostic criteria, including the International Classification of Diseases (ICD-10) and the DSM-IV. It has been validated in Spain [39], and previously has been used in T2DM patients [40], and by telephone [41-43].

Personal history of psychiatric disorder will be registered if patient reports a positive response to the question: “A clinician has ever diagnosed you as having any psychiatric disorder? or there is a diagnosis prior to baseline in the in CCR.

The diagnosis of family history of psychiatric disorder will be registered if the patient reports a positive response to the question: “Has any family member (in first-degree relatives) ever been diagnosed of psychiatric disorder?”

Social support will be measured by the question: “About how many close friends and close relatives do you have (people you feel at ease with and can talk to about what is on your mind)?”, which corresponds to the first item of the Medical Outcomes Study-Social Support Survey (MOS-SSS) [44].

To assess the HRQoL, the Medical Outcomes Study 12-Item Short-Form Health Survey (SF-12) [45] will be used. SF-12 is used as a generic measurement of global functioning and HRQoL, is non-disease specific and comprises 12 questions about the physical health component (physical functioning and role limitations due to physical health) and the mental health component (bodily pain, vitality, social functioning, role limitations due to emotional problems, and mental health).

The self-reported of sleep quantity will be measured by the question: “What was the average amount of sleep per night (in hours) and per day (in hours), during the previous month?”. Sleep quality will be measured by the Athens Insomnia Scale (AIS) [46]. The AIS is a self-assessment psychometric instrument designed for quantifying sleep difficulty, and its consistency, reliability, and validity has been ascertained as a screening or diagnosis tool for insomnia.

### Assessment of vital status and death cause

Vital status and death cause will be obtained using data collected by general practitioners, by the telephone interview and it will be ascertained using confidential record linkage with the Spanish National Death Index.

### Loss to follow-Up

A low “lost to follow-up rate” will be essential for the validity of the study. The total loss to follow-up at the end of the study should be kept at less than 10% of the recruited population.

In order to minimize the losses to follow-up attributable to general practitioners whom do not continue in the study clinical data will be provided from CCR of patients.

If any patient changes of domicile, an official address search via the local health administration will be conducted.

In order to minimize losses attributable to a fail in locate the patient, up to six telephone calls will be made at different times and on different days; after six failed calls, will try to contact the patient by the general practitioners.

### Statistical analysis

The following analyses will be undertaken:

Descriptive analysis of each variable. Description of the profile of patients who abandon the study plus their reason for withdrawal.

Comparison between depressed and not depressed patients with regards to response variables, and prediction factors. Bivariate statistical tests will be used suitable to the type of variable (qualitative or quantitative).

Analysis of primary outcome. There will be a comparison between depressed and not depressed patients in all-cause and cardiovascular mortality, acute myocardial infarction and stroke using the Chi-squared test.

Logistic regression with random effects will be used to adjust for prognostic factors. Confounding factors that might alter the effect recorded will be taken into account in this analysis.

To assess the effect of depression on the mortality, a survival analysis will be used comparing the two groups using the log-rank test. The control of potential confounding variables will be performed by the construction of a Cox regression model.

All analyses will be calculated with their 95% confidence interval; statistical significance will be set at p<0.05. Statistical processing of the data will be performed with SPSS1 v.18 software.

### Ethical considerations

The study protocol was approved by the Research Ethics Committee of the Carlos III Hospital in Madrid and met all good clinical practice demands rights.

## Discussion

The Spanish National Health System offers coverage to over 95% of the population [47] and drugs prescribed are partially or wholly financed, chronic patients normally visiting PCHC to receive prescriptions; for this reason we consider that the proportion of patients who may not have been included in the randomization of our study to be low.

On the other hand, our work may have a selection bias associated with the participation of both health professionals and patients volunteers.

Our study’s main contribution is to evaluate the increase in the risk of cardiovascular morbidity and mortality, in depressed Spanish adults with T2DM attended in Primary Health Care Setting.

If it is shown that depression is a risk factor that increases morbidity and mortality in patients with T2DM, would be useful to have a predictive clinical model to emphasize our attention in those patients with T2DM that were more prone to depression, acting in a timely manner to prevent or treat early.

Considering the size of the population that could be affected by these two prevalent disorders, further consideration is required to design strategies aimed to provide adequate psychological management and support among those with longstanding diabetes and depression.

The results will be transmitted in the short term to clinicians, so that the results will be integrated into the normal practice in management of patients with T2DM.

## Abbreviations

AIS: Athens Insomnia Scale; CCR: Computerized Clinical Records; DSM-IV: Diagnostic and Statistical Manual of Mental Disorders-Fourth Edition; eCRF: Electronic Case Report Forms; HRQoL: Health related quality of life; ICD-10: International Classification of Diseases; MINI: Mini-International Neuropsychiatric Interview; MOS-SSS: Medical Outcomes Study-Social Support Survey; OR: Odds Ratio; PHCC: Primary Health Care Centres; SF-12: Short-Form Health Survey; T2DM: Type 2 Diabetes Mellitus.

## Competing interests

The authors declare that they have no competing interests.

## Authors’ contributions

CBL conceived the study. MASF and PGC participated in its design and coordination. JCV, CYFR, MIGM and FLL collaborated in the methodology and the bibliographical search. CBL, PGC and MASF drafted the manuscript. Contributions were made by the remaining authors. All authors have read and approved the final manuscript.

## Authors information

MADIABETES Group

A M Alayeto-Sánchez, B Álvarez-Embarba, A M Arias-Salgado-Robsy, A Arnaiz-Kompanietz, E Barrios-Martos, D Beamud-Victoria, M J Bedoya-Frutos, C Bello-González, M Caballero-Sánchez, M E Calonge-García, E Calvo-García, M Camarero-Shelly, A Cano-Espín, P P Carreño-Freire, C Casella-Barban, M J Castillo-Lizarraga, J Castro-Martín, M A Cava-Rosado, I Cerrada-Somolinos, R de Felipe-Medina, G de la Fuente-de la Fuente, S de la Iglesia-Moreno, B de Llama-Arauz, A de Miguel-Ballano, M de Vicente-Martínez, M A Díaz-Crespo, M Domínguez-Paniagua, E M Donaire-Jimenez, J Escobar-Moreno, J Fernández-García, M R Fernández-García, E Fonseca-Capdevilla, F García-García, J N García-Pascual, P Gil-Díaz, M Gil-Díaz, M J Gomara-Martínez, M S Gomez-Criado, E Gomez-Navarrro, A González-González, M I González-García, P Huellin-Martín, J Innerarity-Martínez, Á Jaime-Siso, B E León-Morales, E López-Parra, C López-Rodríguez, M B López-Sabater, A Maestro-Martín, M Martín-Bun, M R Martín-Cano, C Martín-Madrazo, J Martínez-Irazusta, F Mata-Benjumea, A I Menéndez-Fernández, T Mesonero-Grandes, M Miguel-Garzón, C Montero-Lizana, M C Montero-García, A Montilla-Bernabé, A Moran-Escudero, A Muñoz-Cildoz, S Muñoz-Quiros-Aliaga, E Muro-Díaz, S Núñez-Palomo, O Olmos-Carrasco, M C Ortega-Huerta, M E Pejenaute-Labari, M A Pellus-Pardines, E Peña-Rodríguez, F C Pérez-Sánchez, N Pertierra-Galindo, A Pinilla-Carrasco, A Pozo-Teruel, M P Puebla-Sanz, S Pulido-Fernández, A B Ramírez-Puerta, G Reviriego-Jaén, C Reyes-Madridejos, P Ríus-Fortea, G Rodríguez-Castro, M A Rodríguez-Posada, J Roldan-San Juan, M T Rollan-Landeras, A Rosillo-González, C Ruiz-Tuñón, M T Salamanca-Sánchez-Escalonilla, F J San Andrés-Rebollo, J M San Vicente-Rodríguez , M M Sanz-Pascual, L Serrano-González, P Serrano-Simarro, D Serrano-Tomat, M E Serrano-Serrano, A M Sobrado-de Vicente-Tutor, J Suero-Palancar, T Torices-Rasines, P Tovar-García, M A Usero-Martín, E Vaquero-Lucas, I Vázquez-Burgos, B Vázquez-Rodríguez, M P Vich-Pérez, M M Zamora-Gómez, M P Zazo-Lázaro.

## Pre-publication history

The pre-publication history for this paper can be accessed here:

http://www.biomedcentral.com/1471-244X/12/95/prepub
